# Aquaporin 8ab is required in zebrafish embryonic intestine development: AQP8 in zebrafish embryonic intestine development

**DOI:** 10.3724/abbs.2022077

**Published:** 2022-07-12

**Authors:** Shiping Wang, Yinyin Qin, Jiajing Sheng, Xuchu Duan, Lizong Shen, Dong Liu

**Affiliations:** 1 Division of Gastrointestinal Surgery Department of General Surgery the First Affiliated Hospital Nanjing Medical University Nanjing 210029 China; 2 The Affiliated Suqian First People’s Hospital of Nanjing Medical University Suqian 223800 China; 3 School of Life Science Co-innovation Center of Neuroregeneration Key Laboratory of Neurogeneration of Jiangsu and MOE Nantong 226001 China

**Keywords:** aqp8ab, gastrointestinal tract, intestine development, intestinal lumen, zebrafish

## Abstract

The aquaporin 8 (AQP8) is a small integral membrane protein that selectively transports water and other small uncharged solutes across cell plasma membranes. It has been demonstrated that AQP8 is ubiquitously present in various tissues and organs of mammals, and participates in many physiological and pathological processes. Recent studies showed that AQP8 is highly expressed in the columnar epithelial cells of mammalian colonic mucosa facing lumen, indicating that AQP8 plays potential roles in the physiology and pathophysiology of gastrointestinal tract. However, the role of AQP8 during gastrointestinal tract development is unclear. In the present study, RT-PCR results reveal that the zebrafish genome encodes three kinds of *aqp8s* ( *aqp8aa*, *aqp8ab*, and *aqp8b*). We use whole mount in situ hybridization to describe *aqp8* genes spatiotemporal expression pattern, and the results show that *aqp8ab* mRNA is detectable mainly in the zebrafish embryonic intestine. To reveal the details of *aqp8ab* distribution, histological sections are employed. Transverse sections indicate that *aqp8ab* mRNA expression is more intense in the layer lining the intestinal cavity. Knockout of *aqp8ab* using the CRISPR/Cas9 system induces intestine development defects and abnormal formation of intestinal lumen. In addition, *aqp8ab* mRNA significantly rescues the intestine defects in the *aqp8ab* mutant. These results indicate that *aqp8ab* is required in the intestine development of zebrafish.

## Introduction

The primary functions of the gastrointestinal (GI) tract are digestion, absorption, excretion, and protection [1]. To perform these functions, the GI epithelium in close contact with the GI lumen forms a physical and biochemical barrier to separate the GI lumen from the underlying tissues [ 2– 4] . Water, electrolytes and nutrients move across the barrier in a regulated manner either between epithelial cells or across the apical membrane of epithelial cells. Proper function of the GI tract is essential for supporting life. A growing body of evidence suggests that defects in intestinal barrier function are associated with several different human diseases, including inflammatory bowel disease (IBD), diarrhea, celiac disease, and irritable bowel syndrome (IBS) [5]. However, the mechanisms responsible for GI physiology and function and the critical steps in GI disease pathogenesis are still unclear.

Transcellular water flux primarily occurs via aquaporins (AQPs) in the mammalian digestive system [6]. The AQPs, which belong to major intrinsic protein (MIP) family, are integral membrane proteins that selectively transport water and other small uncharged solutes across cell plasma membranes [ 7- 9] . Until now, at least eight AQPs have been found to be expressed in various tissues in the mammalian GI tract, and they are distributed in a distinct regional and subcellular manner [ 10– 12] . The special locations of AQPs are relevant to their functions. Numerous studies have demonstrated that the alterations of AQPs expression and distribution in the GI are associated with gut disorders and diseases [ 13– 15] .

It has been demonstrated that AQP8 is highly expressed in the columnar epithelial cells of mammalian colonic mucosa facing lumen, indicating that AQP8 plays potential roles in the physiology and pathophysiology of gastrointestinal tract [ 16– 19] . In contrast to mammals, three kinds of *aqp8s* ( *aqp8aa*, *aqp8ab*, and *aqp8b*) are have been reported in zebrafish [ 20, 21] . The *aqp8s* have been found to be expressed in the digestive system of zebrafish and possibly play critical roles in controlling or maintaining water homeostasis. However, the role of zebrafish *aqp8s* in GI organ morphogenesis throughout the embryonic development remains unclear.

Previous studies have shown that intestinal anatomy and architecture in zebrafish is closely related to mammals, and it has emerged as a valuable model organism for genetic research of vertebrate organ morphogenesis [ 22– 26] . The zebrafish intestinal tract is divided into three main segments: the intestinal bulb, the mid-intestine and the posterior intestine. Instead of a stomach, the intestinal bulb acts as the major site of lipid and protein digestion [27].

In the present study, we used the zebrafish model to analyze the role of *aqp8s* in intestine development. RT-PCR and whole mount *in situ* hybridization results revealed that *aqp8aa* was highly expressed in the vascular system, *aqp8ab* was observed in the intestine, and *aqp8b* was detected in the kidney. To reveal the details of *aqp8ab* distribution, histological sections were employed. Transverse sections indicated that *aqp8ab* mRNA expression was more intense in the layer lining the intestinal cavity. Knockout of *aqp8ab* using CRISPR/Cas9 system induced intestine development defects and abnormal formation of intestinal lumen. In addition, *aqp8ab* mRNA significantly rescued the intestine defects in the *aqp8ab* mutant. These results indicate that *aqp8ab* is required in the intestine development of zebrafish. Furthermore, our results further promote the understanding of the cellular and molecular mechanisms responsible for GI organ morphogenesis and patterning.

## Materials and Methods

### Zebrafish tissues and embryos

The zebrafish (AB line) used in this study was obtained from China Zebrafish Resource Centre (Wuhan, China) and maintained in Jiangsu key laboratory of Neuroregeneration of Nantong University (Nantong, China). Zebrafish embryos were obtained through natural mating and maintained at 28.5°C. Embryonic stages were defined as described previously [28]. At 24 h post-fertilization (hpf), embryos were treated with 0.2 mM 1-phenyl-2-thio-urea (PTU), a tyrosinase inhibitor commonly used to block pigmentation and aid visualization of zebrafish development. Embryos for whole mount *in situ* hybridization (WISH) were collected at various stages, fixed with 4% paraformaldehyde (PFA) in phosphate-buffered saline (PBS) overnight at 4°C or 2 h at room temperature, washed with PBST (PBS plus 0.1% Tween-20), dehydrated in methanol, and stored at –20°C until use. Embryo development stages less than 24 hpf were dechorionated after fixation, prior to storage. For zebrafish over 4 days post-fertilization (dpf), immobilization by submersion in ice water (5 parts ice/1 part water, 0–4°C) for at least 20 min to ensure death by hypoxia. For embryos ≤ 3 dpf, development was terminated using bleach. Addition of bleach solution (sodium hypochlorite 6.15%) to the culture system water at 1 part bleach to 5 parts water. The embryos remained in this solution at least five minutes prior to disposal to ensure death. This procedure was approved by the Administration Committee of Experimental Animals of Nantong University (Approval ID: 20180608-Z001). The pain perception has not developed at these earlier stages, so this is not considered a painful procedure. All animal experiments were carried out in accordance with the NIH Guidelines for the Care and Use of Laboratory animals ( http://oacu.od.nih.gov/regs/index.htm). This study covers the 3Rs (refinement, replacement, and reduction).

### RNA extraction, reverse transcription, and PCR

Embryos and tissues were homogenized and frozen using Trizol reagent (Invitrogen, Carlsbad, USA) and stored at –80°C. Total RNA was extracted according to the manufacturer’s instruction. RNA (1 μg) was reverse-transcribed into cDNA using Transcriptor First Strand cDNA Synthesis kit (Roche, Basel, Switzerland) according to the manufacturer’s instruction. Synthesized cDNA was stored at –20°C. All PCR amplifications were carried out in a total volume of 50 μL using 2× *Taq* PCR SuperMix (Trans, Beijing, China) according to the manufacturer’s instruction.

### Whole mount *in situ* hybridization

The 501 bp coding sequence for zebrafish *aqp8aa* (Ensemble Transcript ID: ENSDART00000066382.6) was amplified by PCR using the following primers: left primer, 5′-TTCAGCAATGCAACAGGAGC-3′; and right primer, 5′-GGCGAAGAGACATTTAAGCATC-3′. The 504 bp coding sequence for zebrafish *aqp8ab* (Ensemble Transcript ID: ENSDART00000105952.4) was amplified by PCR using the following primers: left primer, 5′-ATCCCATTTCAACCCTCCGT-3′; and right primer, 5′-ACCCGTAGCTTTTCATCTCCA-3′. The 549 bp coding sequence for zebrafish *aqp8b* (Ensemble Transcript ID: ENSDART00000122968.3) was amplified by PCR using the following primers: left primer, 5′-TGATGGGCTGTTTGTGTGTG-3′; and right primer, 5′-CCACCCAGTAGATCCAGTGG-3′. Digoxigenin (DIG)-labeled RNA sense and antisense probes were made from the linearized plasmids using the DIG RNA Labeling kit (SP6/T7; Roche) according to the manufacture’s protocol. The WISH procedure was modified from these previous *in situ* hybridization protocols [29]. The small baskets were not used in our protocol. BM purple AP substrate (Roche) was used instead of the staining solution. Boehringer blocking reagent (BBR; Roche) was used for blocking.

### Guide RNA (sgRNA) and *Cas9* mRNA preparation

The sgRNA targeting the third exon of *aqp8ab* was designed by using the CRISPR online tool ( https://zlab.bio/guide-design-resources). Effective targeting site is: 5′-GGTGACTCTGGTGGTCCTGA-3′. The mRNA of *aqp8ab* sgRNA and *Cas9* were synthesized *in vitro* using the MAXISCRIPT T7 kit (Ambion, Austin, USA) and mMESSAGE mMACHINE SP6/T7 kit (Ambion), respectively. Then the mRNA was purified using the RNeasy Mini kit (Qiagen, Hilden, Germany), and dissolved in RNase free Ultapure water (Thermo Fisher Scientific, Waltham, USA).

### Injection of mRNAs and identification of *aqp8ab* mutant

To generate mutants, *aqp8ab* sgRNA (500 ng) and *Cas9* mRNA (1500 ng) were co-injected into zebrafish embryos at the 1-cell stage, and after injection of mRNAs, embryos were maintained in E3 medium at 28.5°C. At 24 hpf after mRNAs were injected, 10–20 embryos were collected and their genomic DNAs were extracted. The genomic region surrounding the *aqp8ab* sgRNA targeting site was amplified by PCR. PCR products were subcloned into the pGEM-T Easy vector (Promega, Madison, USA) and were subject to Sanger sequencing. The primers used for PCR and Sanger sequencing are listed as follows: PCR forward primer: 5′-CGATGGTTGTCCCGTATCTT-3′, reverse primer: 5′-AGTGAACGTGCGTACATGCT-3′; and sequencing forward primer: 5′-GTTATGACTTCAGATGAAAA-3′, reverse primer: 5′-CCTGCTAGAATATTGACAAT-3′.

### *aqp8ab* mRNA preparation and injection

The cDNA containing the coding sequence of the *aqp8ab* gene was cloned into PCS2+ vector and then was transcribed *in vitro* using the mMESSAGE mMACHINE SP6 kit (Ambion) after the recombinant plasmid linearized with *Not*I (NEB, Beverly, USA), and then the capped mRNA was purified using the RNeasy Mini kit (Qiagen). *aqp8ab* gene mRNA (2 nL) was injected at 50 ng/μL into the 1-cell stage embryos.

### Histology

For sectioned histological analysis, the whole-mount *in situ* hybridized embryos in 100% glycerin were replaced by Tissue-Tek OCT (Sakura, Tokyo, Japan) two times for 30 min each to remove the glycerin. Subsequently, embryos were embedded in plastic molds containing OCT and the embryos orientation was adjusted with a needle. The embryos were then sectioned with a CM1900 UV (Leica, Wetzlar, Germany) at 14 μm. After being dried for 4 to 5 h at 37°C, the sectioned samples were washed three times (10 min each) with PBS and stored with a mounting medium.

### Microscopy analysis

The results of whole-mount *in situ* hybridization and pictures in bright field were documented with an Olympus DP60 camera on an Olympus stereomicroscope MVX10 (Olympus, Tokyo, Japan), Leica imaging system on a Leica compound microscope and Zeiss SteREO Discovery V20 microscope with a Zeiss AxioCam HRc camera (Zeiss, Oberkochen, Germany). For confocal imaging of intestine development in zebrafish embryos, they were anesthetized with egg water (0.16 mg/mL tricaine/1% 1-phenyl-2-thiourea; Sigma, St Louis, USA) and embedded in 0.6%–0.8% low melting agarose. Confocal imaging was performed with a Nikon TI2-E-A1 HD25 laser scanning confocal microscope (Nikon, Tokyo, Japan).

### Statistical analysis

Image analysis was performed using Imaris microscopy image analysis software (Bitplane AG, Zurich, Switzerland). Statistical analysis was performed using GraphPad Prism (GraphPad Software, San Diego, USA). Student’s *t*-test was used for comparison between groups, and *P*<0.05 was considered statistically significant.

## Results

### *aqp8ab* is expressed in developing intestine of zebrafish

To determine the role of *aqp8ab* in the GI development, we investigated the expression of *aqp8ab* during embryonic development in zebrafish via WISH using antisense RNA probe. We successfully cloned three kinds of *aqp8s* ( *aqp8aa*, *aqp8ab*, and *aqp8b*) using RT-PCR from zebrafish embryos. To determine the tissue-specific expression pattern of *aqp8s*, we performed WISH analysis using digoxigenin (DIG)-labeled antisense probe. The results showed that *aqp8aa* was highly expressed in vascular system, *aqp8ab* was observed in the intestine, and *aqp8b* was detected in the kidney ( Supplementary Figure S1).

**Figure 1:**
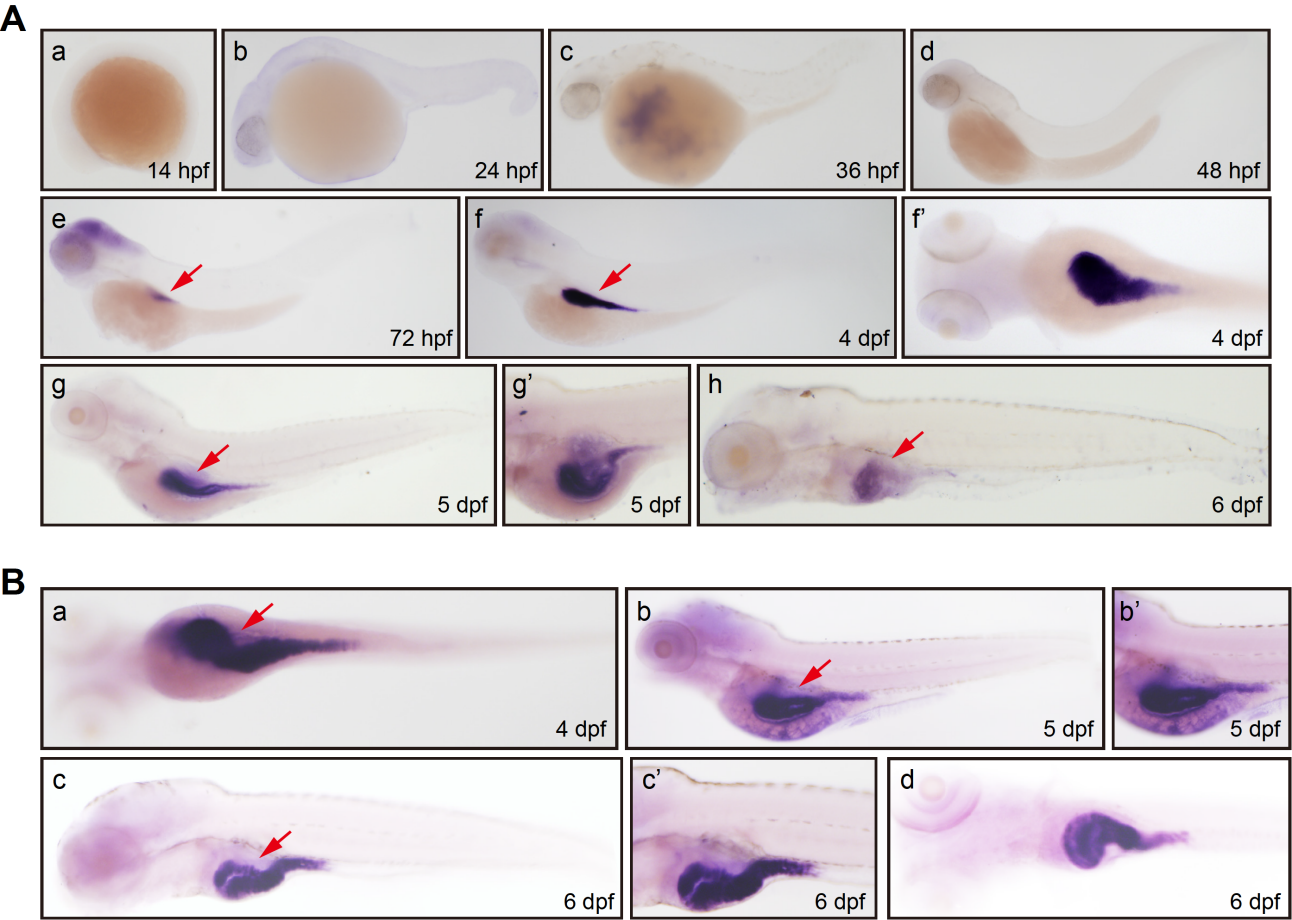
Figure 1 *aqp8ab* and *ifabp* expressions in zebrafish during embryonic development (A) Whole mount in situ hybridization was used to detect the spatial expression of aqp8ab mRNA. (A, part a) 14 hpf, lateral view, no staining. (A, part b) 24 hpf, lateral view, no staining. (A, part c) 36 hpf, lateral view, no staining. (A, part d) 48 hpf, lateral view, no staining. (A, part e) 72 hpf, overview of whole body, intestine (arrow). (A, part f) 4 dpf, overview of whole body, intestine (arrow). (A, part f’) 4 dpf, ventral view, intestine. (A, part g) 5 dpf, overview of whole body, intestine (arrow). (A, part g’) 5 dpf, lateral view, intestine. (A, part h) 6 dpf, overview of whole body, intestine (arrow). (B) Whole mount in situ hybridization analysis was used to detect ifabp mRNA expression in different stages of zebrafish embryonic development. (B, part a) 4 dpf, ventral view of whole body, intestine (arrow). (B, part b) 5 dpf, lateral view, intestine (arrow). (B, part b’) 5 dpf, lateral view, intestine. (B, part c) 6 dpf, overview of whole body, intestine (arrow). (B, part c’) 6 dpf, lateral view, intestine. (B, part d) 6 dpf, ventral view, intestine.

To investigate the expression dynamics of *aqp8ab* during embryonic development, we performed detailed WISH analysis using *aqp8ab* probe. In the 14, 24, 36 or 48 hpf stage embryo, the hybridization signal of *aqp8ab* was not detected. In the 72 hpf stage embryo, weak expression of *aqp8ab* was observed in zebrafish intestine ( Figure 1A). The expression of *aqp8ab* in intestine was increasingly maintained during zebrafish early embryo development. No hybridization signal of *aqp8ab* was found in embryos via WISH using sense RNA probe ( Supplementary Figure S2).

The *aqp8ab* expression patterns were confirmed using the probe of intestinal marker intestine fatty acid binding protein ( *ifabp*; *fabp2-ZFIN)* from Peng’s lab [ 30, 31] , and the result was consistent with the *aqp8ab* probe expression patterns. In the 4, 5 and 6 dpf stage embryos, clear hybridization signal of *ifabp* was seen in zebrafish intestine ( Figure 1B).

### *aqp8ab* mRNA expression is more intense in the layer lining the intestine cavity

**Figure 2:**
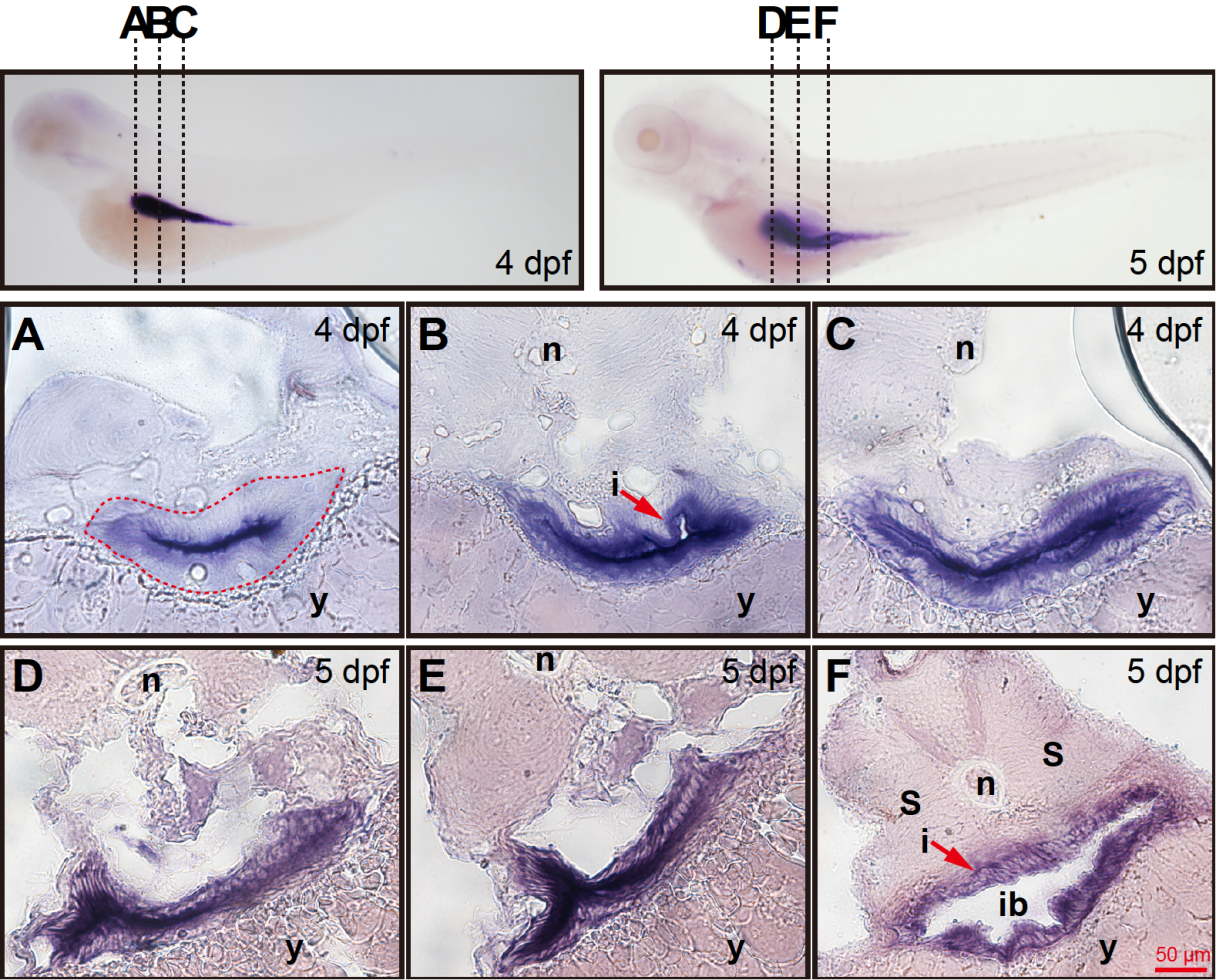
Figure 2 *aqp8ab* mRNA expression is more intense in the layer lining the intestinal cavity (A–C) Corresponding transverse sections through the three different regions of intestine at 4 dpf depicted in left image of the top row. (A) The aqp8ab mRNA was mainly expressed in epithelial cells lining the intestinal cavity. The developing intestine adjacent to the yolk was seen at 4 dpf (red dotted lines). (B) The aqp8ab mRNA expression was more intense in the layer lining the intestinal cavity. The intestinal tract contained a small lumen (arrow). (C) The aqp8ab mRNA expression was still detected in the layer lining the intestinal cavity. (D–F) Corresponding histological cross-sections through the different regions of intestine at 5 dpf depicted in right image of the top row. Cross-sections analysis showed that aqp8ab mRNA expression was more intense in the layer lining the intestinal lumen. n, notochord; i, intestine; s, somite; y, yolk; ib, intestinal blub.

To reveal the details of *aqp8ab* distribution in the zebrafish intestine, we conducted histological section experiments on embryos. Transverse sections showed that in the anterior and middle region of the intestinal tract at 4 dpf, the *aqp8ab* mRNA expression was presumably confined to the epithelial cells lining the intestinal cavity ( Figure 2A,B). By 5 dpf, the *aqp8ab* mRNA expression was more intense in the layer lining the intestinal cavity ( Figure 2D–F).

### Establishment of *aqp8ab* knockout mutant line by CRISPR/Cas9 system

**Figure 3:**
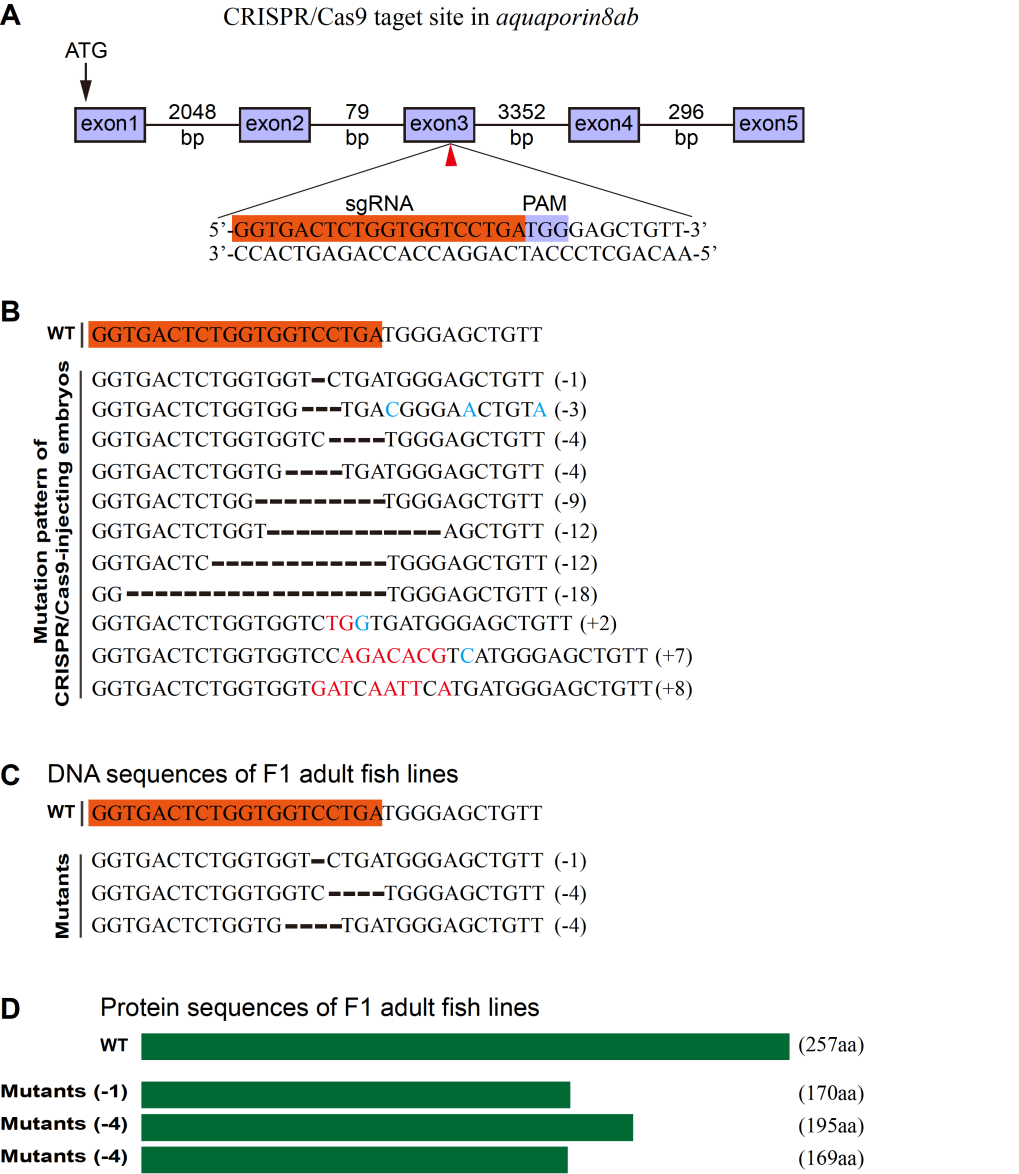
Figure 3 Establishment of *aqp8ab* knockout mutant line by CRISPR/Cas9 (A) Schematic diagram showing aqp8ab sgRNA targeting site (indicated by arrowhead) in the third exon of aqp8ab gene. Starting codon (ATG) site is indicated by arrow. The sgRNA targeting sequence is highlighted in brown and the PAM in purple. (B) Mutation pattern of mRNAs-injected embryos. Numbers in the brackets show the number of nucleotides that were deleted (–) or inserted (+). Inserted nucleotide is in red, changed nucleotide is in blue. WT, wild-type. (C) Three heritable mutants were identified by screening. F0 offspring were out-crossed with wild-type fish to produce F1, and the DNA extracted from tail fins of F1 adults were used for the identification of heritable mutants by sequencing. (D) Schematic diagram showing the predicted proteins encoded by the three mutated alleles. The mutants are reading frameshift mutations that result in truncated proteins.

In order to examine whether *aqp8ab* is required for the development of intestine, the CRISPR/Cas9 system was utilized to generate a series of *aqp8ab* mutants in zebrafish. *aqp8ab* sgRNA mRNA and *Cas9* mRNA were co-injected into the 1-cell stage embryos to knock out the genes. We chose the target sites in the third exon of zebrafish *aqp8ab* and identified the effective sgRNA ( Figure 3A). To examine the mutation type, we extracted genomic DNA from embryos at 24 hpf with injected mRNAs and performed PCR to amplify the target region. PCR products were sequenced and sequence analysis revealed 11 types of mutations ( Figure 3B). The remaining sibling of these G0 embryos was raised to adulthood. The G0 founders carrying somatic mutations were out-crossed with wild-type fish to obtain F1 offspring. We identified 3 types of mutations among the adult F1 fish by PCR amplification and sequencing with tail fin-clipped genomic DNAs ( Figure 3C). The mutated alleles included a 1-bp deletion, a 4-bp deletion and another 4-bp deletion, which all resulted in reading frame shift and premature translation termination ( Figure 3D). According to the previous protein sequences analysis, the truncated proteins lose three essential domains including NPA, H7 and H8 [20]. Moreover, the abnormal mRNAs will probably be degraded by the mechanism of NMD, which results in no protein production.

### *aqp8ab* deletion induces abnormal phenotype in embryonic development

**Figure 4:**
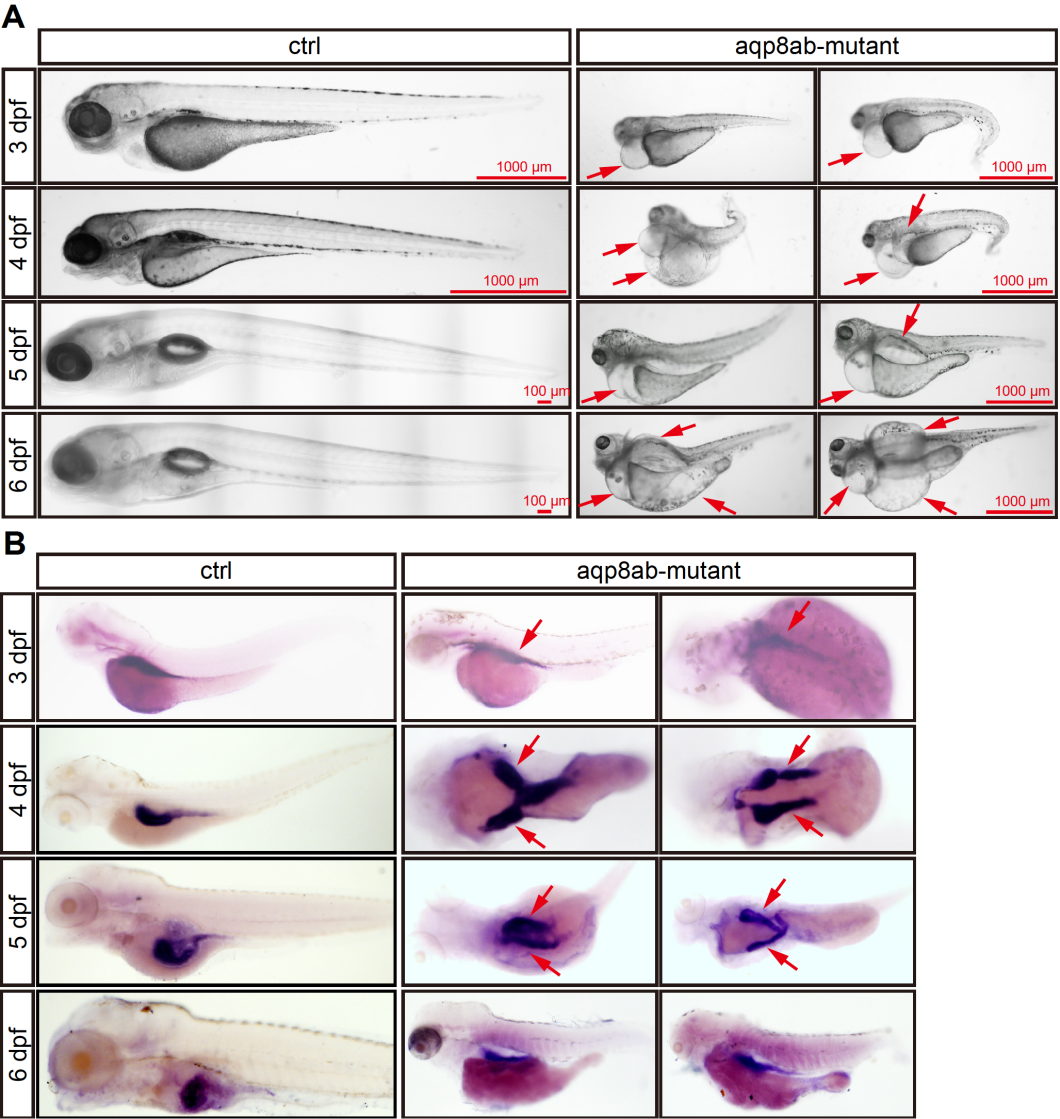
Figure 4 Effect of *aqp8ab* deletion in zebrafish embryonic intestine development (A) Microscopy analysis of embryos development at 3–6 dpf in control group and aqp8ab mutants. Red arrows indicate pericardial edema. (B) Whole mount in situ hybridization detection of the expression of ifabp at 3–6 dpf after aqp8ab deletion. Red arrows indicate intestinal bifida.

To examine the effect of the mutation during intestine development, we observed embryo development at different stages in bright field. The deletion of *aqp8ab* had no significant effect on embryonic development in the early stage (data not shown). At stage 3 dpf, the embryo sizes of the *aqp8ab* mutants were decreased and had slight pericardial edema phenotype ( Figure 4A). At 4 and 5 dpf, the deletion of *aqp8ab* induced severe shortening of the body axis, and pericardial edema. More than 47% (40/85) *aqp8ab* mutants had serious edema phenotype at 6 dpf.

To detect the effects of *aqp8ab* deletion on the intestinal morphology, we carried out WISH using *ifabp* probe. The mutants had a normal intestinal tract at 3 dpf ( Figure 4B). At 4 and 5 dpf, compared with the control group, the deletion of *aqp8ab* induced zebrafish embryo intestinal bifida, and the intestines were deformed ( Figure 4B). These results suggested that *aqp8ab* might play a role in zebrafish embryonic intestine development.

### Loss of *aqp8ab* impairs zebrafish intestinal lumen formation

**Figure 5:**
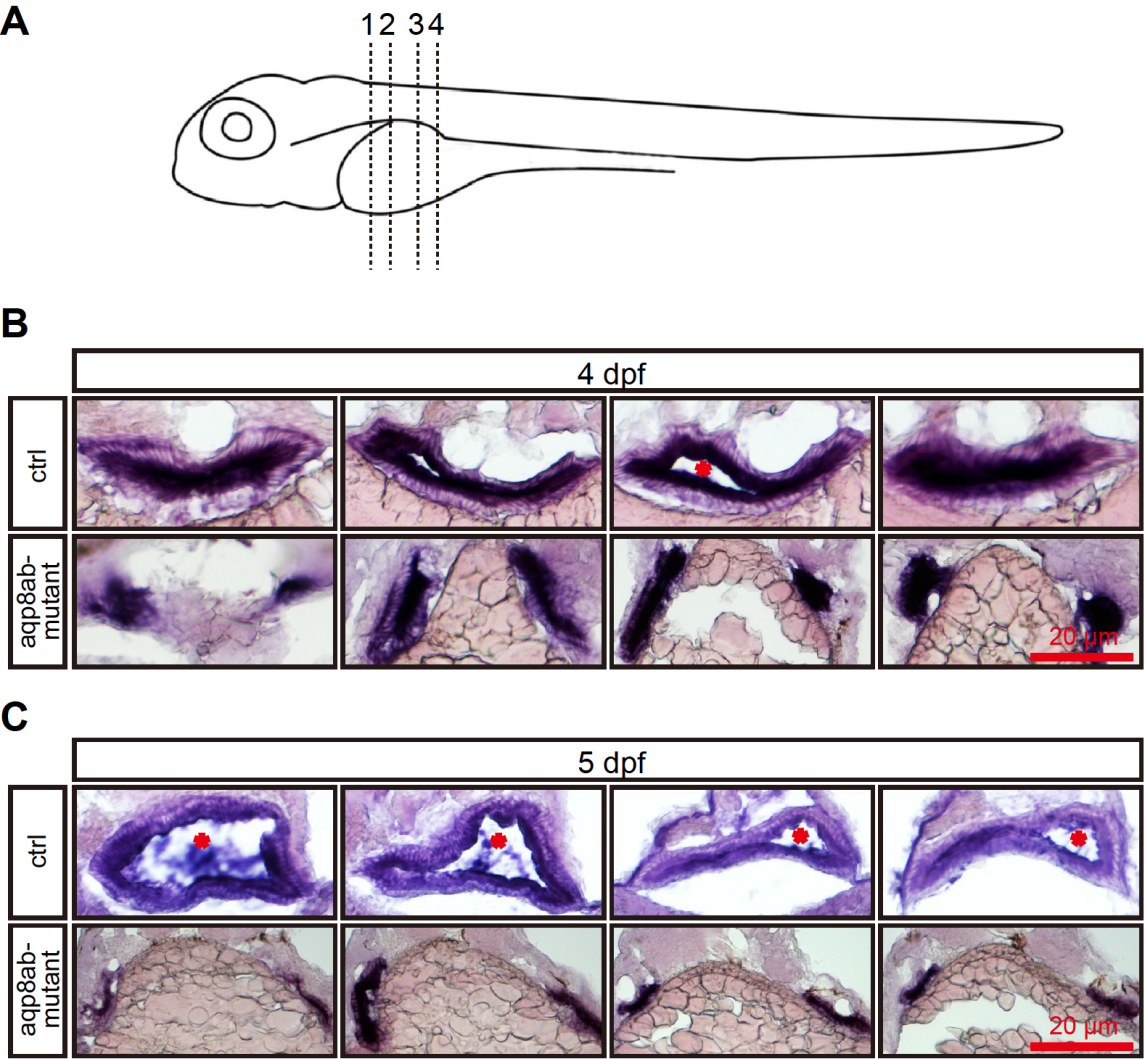
Figure 5 Loss of *aqp8ab* impairs zebrafish intestinal lumen formation (A) Schematic diagram showing transverse sections through the trunk at four different regions. (B) At 4 dpf, the control group showed a single intestinal tract, in which a lumen was already formed (stars). The aqp8ab mutants displayed double different intestinal tracts with no lumen. (C) At 5 dpf, the diameter of intestinal tract in the control group grew bigger. The aqp8ab mutants showed double different intestinal tract with no lumen.

To further detect the function of *aqp8ab* in intestine development, the embryos after WISH were sectioned ( Figure 5 and Supplementary Figure S3). In the control group, transverse sections through the trunk at four different regions demonstrated a single intestinal tract, in which a lumen was already formed ( Figure 5B,C). Cross-sections revealed double independent intestinal tracts with no lumen or a small lumen at 4 and 5 dpf, compared with the control group ( Figure 5B,C and Supplementary Figure S3). These results suggested that embryonic intestinal lumen development might be impaired.

### *aqp8ab* mRNA injection can rescue the abnormal phenotype induced by *aqp8ab* gene deficiency

**Figure 6:**
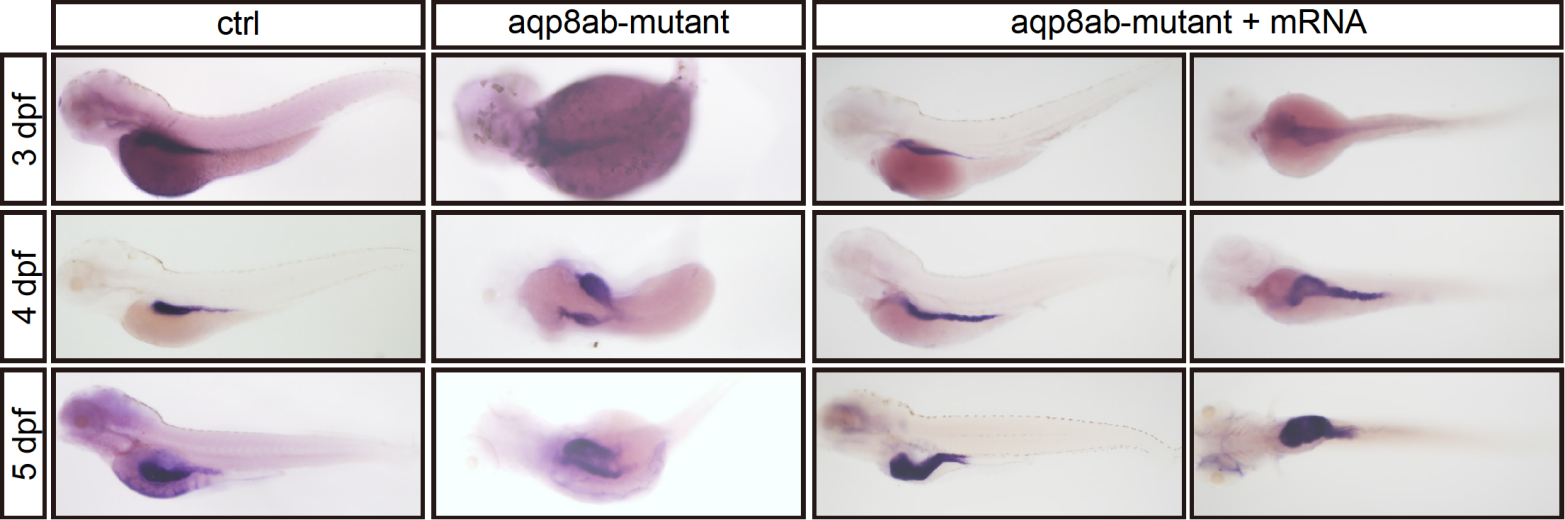
Figure 6 *aqp8ab* mRNA injection can rescue the abnormal phenotype induced by *aqp8ab* deficiency At 4–5 dpf, the control group had a single intestinal tract, the aqp8ab mutants displayed double different intestinal tracts. However, aqp8ab mRNA injection could rescue the phenotype caused by aqp8ab mutation. In the rescue experiment, left embryos shown here are lateral view with anterior to the left, and right embryos shown here are ventral view with anterior to the left.

To further determine whether the abnormal phenotype observed in the *aqp8ab* mutants is due to the loss of function of *aqp8ab* rather than to nonspecific effects, we performed the rescue experiment. The mixture of sgRNA (500 ng), *Cas9* mRNA (1500 ng) and *aqp8ab* mRNA (250 ng) were injected into the 1-cell stage embryos. It was found that the embryos co-injected with *aqp8ab* mRNA can rescue the intestine development defects ( Figure 6). These results showed that intestine defects are specifically caused by inactivation of *aqp8ab*.

## Discussion

AQPs are a group of membrane transport proteins that assemble in cell plasma membranes as tetramers and facilitate the passage of water and other small solutes, such as glycerol, urea, and carbon dioxide. To date, 13 mammalian AQPs have been identified, which are present in various tissues and organs with functions including from acid secretion [32], renal collection ducts and body water homeostasis [33], blood-brain barrier permeability [34], immune system regulation [35] and tumor biology involvement [36]. AQPs are physiologically essential in mammals.

Previous studies showed that AQP8 is expressed in mammalian digestive system, such as salivary glands, pancreas, liver, small intestine, and large intestine [ 10, 37] . The distributions of AQP8 are relevant to its function, indicating that AQP8 might play potential physiological roles in the digestive system. A recent study revealed that AQP8 facilitates the transport of water and small molecules on the apical membrane of rat small intestine [38]. However, the role of AQP8 during gastrointestinal tract development is still unclear. Here, we cloned three kinds of *aqp8s* ( *aqp8aa*, *aqp8ab*, and *aqp8b*) in zebrafish, and our results showed that *aqp8ab* is essential for intestinal organogenesis. The expression profile of *aqp8ab* was detected, revealing that it is present in various stages during zebrafish embryogenesis and is mainly expressed in the embryonic intestine. The deletion of *aqp8ab* induced intestine development defects and abnormal formation of intestinal lumen.

In a previous study, AQP8 was found to localize to the apical membrane of the colonic intestinal epithelial cells (IECs), and down-regulation of epithelial AQP8 may impair water resorption in active collagenous colitis (CC), resulting in watery diarrhea [19]. The function of AQP8 in the water absorption and secretion of small intestine and colon is limited in *AQP8*–knockout mice model [17]. In the murine colonitis model, the expression level of AQP8 is reversely correlated with the occurrence of inflammation and injury [38]. These data show that the alterations of AQP8 expression may impair the passage of transcellular water and induce inflammatory bowel disease, suggesting that AQP8 is a potential drug target for the treatment of intestinal disorders and diseases.

Intestinal epithelial cells are polarized and form a luminal surface by their apical membranes. In a previous study, the interaction of cortical membrane organizer EMR-1 and AQP8 was found to propel lumen extension by transluminal flux, suggesting a direct morphogenesis effect of water-channel-regulated fluid pressure [39]. The deletion of *aqp8ab* in intestinal epithelial cells impairs the transport of transcellular water and results in the imbalance of water homeostasis, thereby inducing an intestine defect and abnormal formation of intestinal lumen.

In summary, *aqp8ab* affects intestine development in embryos of zebrafish, and the intestinal lumen formation is affected.

## Supplementary Data

Supplementary data is available at *Acta Biochimica et Biophysica Sinica* online.
